# Toward Smart Traceability for Digital Sensors and the Industrial Internet of Things

**DOI:** 10.3390/s21062019

**Published:** 2021-03-12

**Authors:** Sascha Eichstädt, Maximilian Gruber, Anupam Prasad Vedurmudi, Benedikt Seeger, Thomas Bruns, Gertjan Kok

**Affiliations:** 1 Presidential Staff, Physikalisch-Technische Bundesanstalt, 10587 Berlin, Germany; maximilian.gruber@ptb.de (M.G.); anupam.vedurmudi@ptb.de (A.P.V.); 2 Acoustics and Dynamics, Physikalisch-Technische Bundesanstalt, 38116 Braunschweig, Germany; benedikt.seeger@ptb.de (B.S.); thomas.bruns@ptb.de (T.B.); 3 Unit Flow, Van Swinden Laboratory B.V., Thijsseweg 11, 2629 JA Delft, The Netherlands; gkok@vsl.nl

**Keywords:** Internet of Things, calibration, measurement uncertainty, traceability, semantics, ontology, sensor network, digital sensors, redundancy

## Abstract

The Internet of Things (IoT) is characterized by a large number of interconnected devices or assets. Measurement instruments in the IoT are typically digital in the sense that their indications are available only as digital output. Moreover, a growing number of IoT sensors contain a built-in pre-processing system, e.g., for compensating unwanted effects. This paper considers the application of metrological principles to such so-called “smart sensors” in the IoT. It addresses the calibration of digital sensors, mathematical and semantic approaches, the communication of data quality and the meaning of traceability for the IoT in general.

## 1. Introduction

With the increasing use of digital technology, along with large-scale heterogeneous sensor networks and machine learning methods, various new possibilities and challenges have arisen for metrology—the science of measurement [1,2]. The Internet of Things (IoT) as the concept of interconnected devices has only recently been considered in metrology. Of particular importance for metrology is the quantifiable assessment of data quality in any kind of measurement task. For the IoT, data quality is important in all steps in the process from the individual sensing elements to the final step of data processing. For example, in predictive maintenance the quality of the sensor measurements needs to be taken into account to derive a quality statement for the recommendation of maintenance operations. Moreover, measurements with a small measurement uncertainty should be valued higher than others in the data analysis. Therefore, the integration of fundamental metrological principles—traceability to the International System of Units (SI) and the evaluation of measurement uncertainty—into the IoT data life cycle is an area of active research, see, e.g., [1].

A core principle in the IoT is the utilization of sensor networks. That is, the individual measuring instruments are interconnected and form a distributed measuring system. Sensor networks have been investigated for a couple of years in metrology. For instance, in [3] measurement uncertainty evaluation when aggregating data from sensors is considered. The authors in [4] studied the possibility of self-validation in wireless sensor networks from a metrological point of view. The evaluation of uncertainty in sensor networks has been studied mostly for state-space systems, e.g., with the application of Bayesian methods for sensor fusion [5]. As for other areas, the metrological applicability of commonly used mathematical methods has been investigated. For instance, in [6] the authors discuss the relation of the Kalman filter covariance to a measurement uncertainty in line with standards in metrology.

The IoT goes beyond several of the approaches considered in these publications from the metrology community. The size of the heterogeneous sensor networks in IoT scenarios, the data-driven analyses and automated online post-processing of measured data are just some of these differences. Recent research activities in metrology have started to this end. Of particular importance is the traceability of measurements in the IoT to SI units. This is relevant for the comparability of measurements and for improving the reliability and resilience in the IoT. One prerequisite is information about the individual measuring instruments that allows the establishment of a relationship between the indicated values and the actual measurand, e.g., from a calibration. In the IoT often sensors are applied that provide only digital outputs, which is challenging for certain types of calibration. In [7], the authors developed an approach for the traceable calibration of the dynamic behavior of digital sensors. In [1], a batch calibration method for micro-electro-mechanical-systems (MEMS) sensors was developed that utilizes existing MEMS testing equipment.

The IoT is also characterized by a mostly automated flow of information and processing of data. This requires novel approaches for the representation and distribution of calibration data. Therefore, the European research project titled SmartCom [8] addresses the communication of metrological information in digital infrastructures. Our work here extends the recently published work from [8] on digital calibration certificates in sensor networks by an investigation of the potential use and benefits of integrating such metrological information in a sensor network. That is, we consider the availability of information, such as calibration data, and address the utilization of this information to achieve traceability in the IoT.

Traceability in the metrological sense is the relation of a measurement result to a reference through an unbroken chain of calibrations, each contributing to the measurement uncertainty [9]. Therefore, traceability for the IoT begins with the traceability of its measuring devices. In Section 2, the calibration of sensors is considered that also characterizes frequency-dependent behavior, i.e., dynamic calibration. Calibration is of particular importance when measurement results need to be comparable and transferable, e.g., when one measuring device needs to be replaced by another. In addition, fundamental aspects of calibration in sensor networks are discussed in that section. With the increasing use of built-in pre-processing, sensors are becoming “smart” [10] in the sense that they contain a certain amount of pre-processing and awareness about their surroundings. In Section 3, a concept is presented that allows the combination of smart sensors with traceability in the IoT—leading to what can be called “smart traceability”. The proposed concept uses hardware and software elements that can be moved easily between the individual smart sensor, the edge, the fog or the cloud—depending on the available computing power and application. In principle, the utilization of smart sensors is divided into battery and non-battery devices. For sensors that are powered by an internal battery, one needs to balance power consumption and performance. For sensors with an external power supply, such a distinction is not necessary. In this paper, we focus on smart sensors with an external power supply for ease of presentation.

With smart sensors being present in the IoT in reasonable numbers, novel concepts for using semantic information can be applied. In Section 4, an approach is described that utilizes information from smart sensors and other sources to obtain a semantic description of the sensor network. This information is provided in a machine-readable way such that an algorithm can make use of it in the subsequent data analysis. Section 5 outlines the efficient use of the above-described elements for a beneficial application of traceability in the IoT. The detection and utilization of sensor redundancy are therefore considered. A practical example with actual sensor network measurement data is used to illustrate the influence of redundancy. Finally, Section 6 gives some conclusions and an outlook on future developments.

## 2. Dynamic Calibration and Sensor Network Metrology

Even for classic single sensor focused applications, dynamic calibrations were important for knowing how a sensor and the connected measuring chain react to an input quantity varying over time. The basic question here was “Is the sensor agile enough to follow the input?” or in other words “In how far does the sensor’s inertia influence the measurement result?”. The technical answer to this question is the complex transfer function of the sensor which is determined during dynamic calibration. It describes the sensitivity of a sensor in relation to an applied frequency. While the magnitude is what we typically call the sensitivity or gain, the phase describes the delay suffered by a signal from input to output at a given frequency. The frequency dependence of magnitude and phase may already in classic measurements lead to a significant distortion of, for example, transient measurements if the shape of the real input signal and the output of the sensor are compared. Dynamic calibration is a topic of growing interest in metrology and several approaches have been proposed in the literature. The European research project EMRP IND09 (Traceable Dynamic Measurement of Mechanical Quantities) published a best practice guide on the implementation of dynamic calibration (https://www.ptb.de/emrp/ind09-best-practice-guide.html accessed on 30 January 2021).

The typical case considered in metrology so far has been that of sensors whose dynamic behavior can be modeled by a linear time-invariant (LTI) system model. An advantage of LTI systems is that several equivalent representations exist to model the system: the transfer function, frequency response, impulse response and step response. Dynamic calibration then comprises an experiment to determine the model parameters for a suitable representation. For instance, for the transfer function model

(1) $$H\left(s\right)=K\frac{\sum_{m}b_{m}s^{m}}{\sum_{n}a_{n}s^{n}},$$

where *K* denotes the static gain, and $b_{m}$, $a_{n}$ the polynomial parameters of the numerator and denominator, respectively.

**Figure 1:**
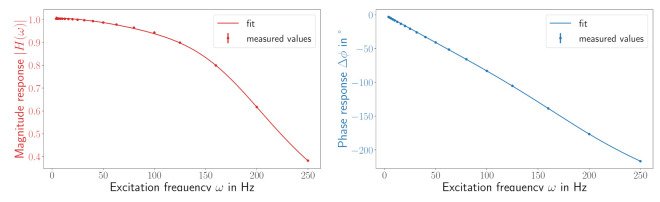
Example: X-axis angular velocity frequency response of an MPU-9250 yielded by dynamic calibration [7]. The frequency response over the calibrated frequency range (4 Hz to 250 Hz) can be described with Equation (1) with K=1 and n,m=8.

Figure 1 shows an example of the dynamic behavior of the x-axis angular velocity sensor in an MPU-9250 inertial measurement unit as a transfer function H(ω) and its model with *K* = 1 and n,m = 8. The transfer function was determined according to the method described in [7] by monofrequent sinusoidal excitation at the one-third octave frequencies according to EN ISO 266 between 4 and 250 Hz with a rotational laser doppler vibrometer as a reference. The polynomial parameters were determined by minimizing the RMS distance of the modeled and measured transfer function coefficients in the complex space using the Broyden–Fletcher–Goldfarb–Shanno (BFGS) algorithm for all possible combinations of 1≤n,m≤10. In sensor networks, in contrast, the focus is not only on a single measurand but usually on the mutual relation of various measurands at different locations. If, for example, inertial sensors are used to track the position and orientation of some larger component by measurements of acceleration or angular rate at different positions on the body, the measurements need to be processed for the same instance in time and the potential distortion of the signal needs to be known in order to compensate it. Therefore, the analysis of the acquired data have to take the individual dynamic calibration of each sensor into account. This is even more important as the increased use of sensor-internal processing power in so-called smart sensors typically leads to a significantly larger delay than the classic analog sensor provides.

Additional complications arise if the application requires information on the individual sample, as for edge-triggering situations. The classic way was to equip the system with a common sample clock or to utilize a centralized digitizer with a common sample clock for all digitized channels. The increasing numbers of IoT sensors are all equipped with their own internal digitizer typically without any means to provide an external common clock. As a consequence the user has to rely on “nominal sampling rates” from the data sheet, without any influence on or detailed knowledge of the accuracy of that internal timing. New calibration methods for digital output sensors can mitigate that problem by external timestamping and comparisons to high-precision laboratory clock facilities. Information about sample frequency deviation or, for example, its temperature drift might be part of a standard dynamic calibration in the future.

The good news is that within a modern IoT network of sensors, it should be simple to manage the distribution of calibration data. A sensor can identify itself to the measurement system master and provide the necessary calibration information once it is connected to the network. This substantially eases the maintenance because the replacement of defective sensors or the amendment of additional sensors becomes simple. That is, with the application of the calibrated model to the sensor output data, an estimate of the measurand, for instance, the sensor input data, can be obtained with associated uncertainties. Another sensor at the same measurement position can be applied in the same way, also providing a means to estimate the value of the measurand with an associated uncertainty. Thus, sensors become replaceable by calibration, and systems become independent of the repeatability of individuals.

This is a huge advantage of the metrological treatment of sensors in the IoT. For a coherent implementation though, measurement uncertainty evaluation in the whole data life cycle may need to be considered. This includes measurement uncertainty evaluation for feature extraction, for signal processing [11] and for the removal of timing effects. Several research activities in these directions are currently in preparation.

## 3. Extending Digital Sensors to Smart Traceability

**Figure 2:**
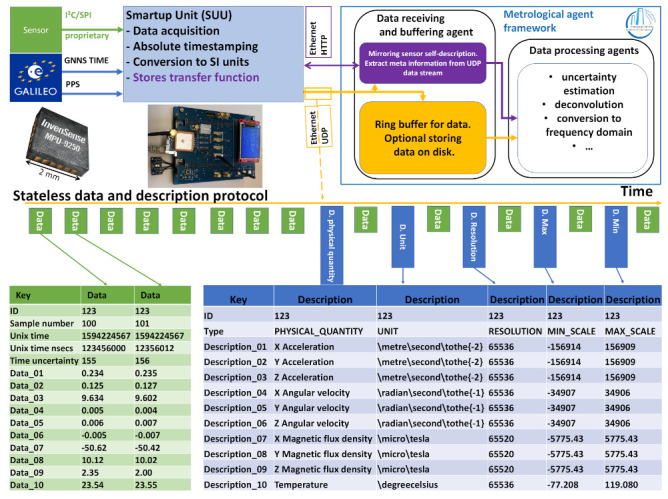
(**Top**) Dataflow from sensor via “Smartup Unit” to a receiving PC running an agent framework for data processing. (**Bottom**) Visualization of stateless data protocol.

We extended a digital sensor with a so-called “Smartup Unit” (SUU), such that the sensor and the SUU together make a smart sensor. The SUU collects the data of the sensors, converts these float values with SI units and provides the data sets with absolute timestamps. At regular time intervals the SUU transmits meta information. This comprises the physical quantity as well as the unit. Furthermore, information about the quantization of the measured values is transmitted, including the minimum and maximum value of the measured quantity, as well as the number of quantization steps. Figure 2 shows the dataflow in the SUU as well as an example of the data yielded from an MPU-9250 inertial measurement unit. Further details on how this can be used to form a semantic self-description will be presented in Section 4. By sending the data to a connected edge device, a mathematical correction of the indicated values can be deployed by using the calibration information contained within the self-description. In the case of a dynamic sensor this could, for instance, be a frequency response characterizing the transfer behavior of the sensor, from which a suitable deconvolution filter is computed and applied. Furthermore, all operations leading to the corrected value need to integrate uncertainty evaluation to maintain metrological traceability. A practical example will be described in more detail at the end of this section.

It is of interest to provide such correction functionality in a form that (1) operates on datastreams with uncertainty, (2) configures itself semi-automatically based on the sensor’s self-description, (3) allows basic building blocks to be reused and (4) hides the complexity of the uncertainty evaluation. A suitable way to achieve these objectives is to make use of the concept of software agents that run on (multiple) edge node(s). Agents and networks thereof represent (physical) objects and actions [12]. Thus, to suit our application, every agent encapsulates a certain processing step which allows for a flexible demand-driven arrangement. Although not implemented here, establishing agent process chains autonomously based on the provided and requested data requirements of the existing agents is feasible. For instance, an interpolating agent only needs to be present in the chain if some later step requires equidistant data. An agent framework that is designed with metrological use cases in mind is the Python package agentMET4FOF 0.4.1 [13]. The following example is implemented using this framework.

As a proof of concept we implement the data correction pipeline for the MPU-9250 sensor used in Figure 1. The code for this example is found in [14]. The pipeline requires the establishment of a connection from the SUU to an edge node, making the stream of sensor data available within the proposed agent framework and computing the semi-automatic correction of indicated sensor values based on their provided self-description. The performance of the implementation is tested up to sampling rates of 6000 Hz. The edge node receives data from the SUU on its network connection. A data-receiving script buffers the incoming data and reports it to programs on request. This interface is used by a source agent to initiate the data stream (using the Python package time-series-buffer) and data description (using the Python package time-series-metadata) within the agent framework. Because the chosen sensor shows a dynamic transfer behavior, we correct the indicated sensor values by the following (manually defined) pipeline: (1) assign an uncertainty to the indicated value if possible (e.g., from quantization information), (2) interpolate the signal to equidistant timestamps, (3) deconvolve the signal with uncertainty using a digital filter computed to match the calibration data and (4) adjust the signal description in every previous step to reflect modifications.

**Figure 3:**
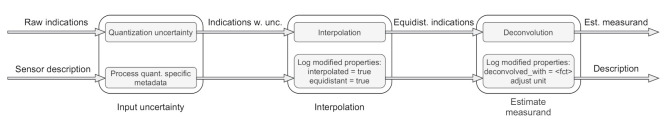
Chain of agents to estimate a measurand using a dynamic calibration model. A generic template for the agents is depicted above the agent chain.

**Figure 4:**
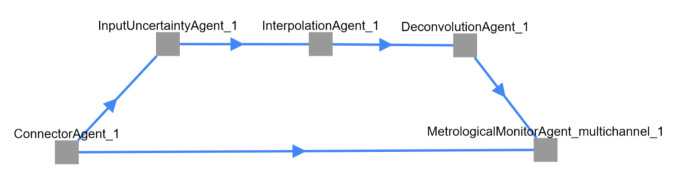
Implemented example: Screenshot of the network topology as shown on the dashboard.

**Figure 5:**
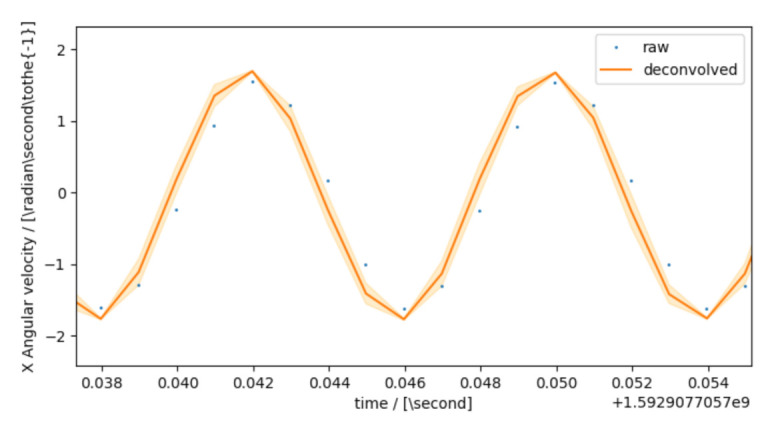
Implemented example: Comparison of the incoming (raw) and processed (deconvolved) signal. Note that the processed signal shows an increase in amplitude that corresponds to Figure 1.

**Figure 6:**
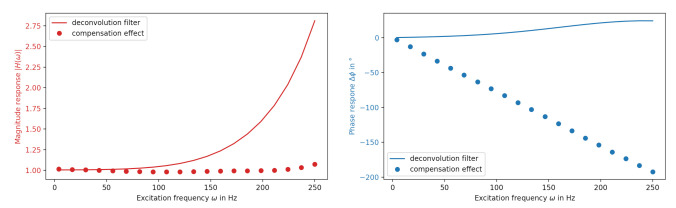
Example: Computed deconvolution filter and compensation behavior of the sensor presented in Figure 1.

The interpolation step is necessary, as common discrete filters expect equidistant streams, but on-board oscillators often deviate from that assumption (which is revealed by the absolute timestamping of our setup). The whole process is visualized in Figure 3, further illustrating the steps behind the top left box of Figure 2. A comparison with the screenshot in Figure 4 shows that every block of Figure 3 represents an actual agent in the implementation. The interpolation and deconvolution routines rely on uncertainty evaluation methods from the Python package PyDynamic 2.0.dev1 [11,15]. We use linear interpolation of uncorrelated time series as published by [16]. This is a rather strong assumption and might lead to unexpectedly low uncertainties of the interpolated signal as well as lower amplitudes of the interpolated signal. Other interpolation schemes will therefore be the focus of further research. The corrected (deconvolved) signal is shown next to the unprocessed input in Figure 5 at a frequency of 125Hz. The filter used to achieve the deconvolution is calculated by a PyDynamic routine by fitting a stabilized inverse filter to the frequency response shown in Figure 1 and is presented in Figure 6. The deconvolution filter is only valid in the same frequency range as the frequency response known from the calibration (and needs to be regularized outside by, e.g., a bandpass). By sticking to a generic agent design, it is possible to implement quite efficient numerical data handling, while also maintaining an expressive degree of data description in every processing step, because both aspects are treated separately.

## 4. Using Semantic Information in Sensor Networks

Semantics, in its most general sense, is the study of meanings in natural or artificial languages. Semantic technologies enable a formal representation of the meaning involved in raw data. Smart sensors are self-aware in the sense that they can provide a self-description. This raises the need for not only machine-readable, but also machine-interpretable representations of asset-specific information and knowledge. Semantic information in sensor networks aims to capture properties of and relations between smart devices. Semantic information is commonly modeled using ontologies [17]. In the context of computer and information sciences, ontologies define a formal representation of a domain of knowledge. The main components of an ontological model are the individual classes belonging to a domain, the attributes or properties of these classes and the relationships among class members.

Semantic information in sensor networks can be modeled by means of ontological structures developed within the semantic web community [18], which formalize the annotation of sensor data with spatial, temporal and thematic metadata [19,20]. Spatial metadata corresponds to information about the location of a sensor, be it according to a geographical or a local reference frame. The latter is particularly relevant to sensors mounted on a moving object like an automobile. Temporal metadata contains information regarding the time instant or interval when the sensor data was recorded. Lastly, thematic metadata provides domain-specific information. In our case, metrologically relevant information would form the bulk of the thematic metadata. A method to merge these kinds of metadata along with the sensor measurement data was proposed in [21]. The main idea of the aforementioned paper is to split the self-description of a sensor into four aspects: (1) observation information, (2) general sensor description, (3) calibration information and (4) location information. Moreover, it is outlined how these aspects can be represented by linking and extending existing schemes, while maintaining metrological requirements. The sensor self-description was achieved by combining existing ontologies that appropriately represented the classes, attributes and relationships corresponding to the aforementioned aspects. In particular, the digital SI (D-SI, [22]) data model was used as the basis to represent the observation values, units and uncertainties. The Semantic Sensor Network (SSN, [23]) and Sensor, Observation, Sampling and Actuation (SOSA, [24]) ontologies enabled the modeling of the core relations between sensors, observations, measurands and measurement procedures. The meta information pertaining to physical quantities, their units and kinds was defined using the Ontology of Units of Measure and Related Concepts (OM, [25]), the Mathematical Markup Language MathML [26] as well as ideas from the Engineering Mathematics (EngMath, [27]) ontology. The semantic structure of the mathematical calibration model was described using MathML, while the Geographic Query Language (GeoSPARQL, [28]) was used to model the geometric and topological location information. The temporal data is represented by the XML “dateTime” datatype in the format “YYYY-MM-DDThh:mm:ss[Z|(+|−)hh:mm]”, where Z refers to the time zone.

**Figure 7:**
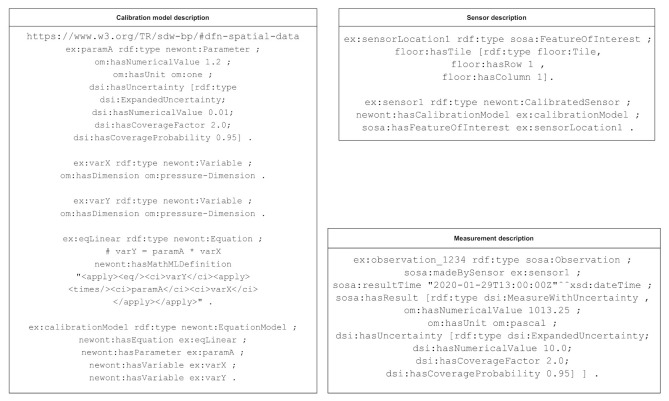
Definitions of the calibration, sensor and measurement models in OWL notation. The prefixes refer to the specific ontologies imported.

The definition of an angular velocity sensor on the MPU-9250 from Section 3 with a calibration model given by the infinite impulse response (IIR) filter Function (1) and a given measurement uncertainty is illustrated in Figure 7 using OWL notation [29]. The definition was adapted from the simpler case of a pressure sensor with a linear calibration model given in [21]. The numerical values, units and dimensions of the variables and parameters are defined using the OM ontology with a prefix om. The measurement uncertainty and its associated parameters like the coverage factor and coverage probability are defined using the digital SI model with the prefix dsi. The metadata corresponding to the sensor description is described using the SOSA ontology with the prefix sosa. The SUU is the platform which hosts the sensor and is represented by the sosa:platform class. The prefix newont is used to denote the ontology resulting from the combination of the aforementioned models. The classes necessary to describe calibrated sensors are defined as part of this ontology. The object ex:calibrationModel is defined using Equation (1) as an instance of the newly defined newont:continuousIIRModel class of the newont ontology with parameters ex:paramA, ex:paramB and ex:paramK. While ex:paramK is a scalar parameter with an uncertainty of type dsi:ExpandedUncertainty, the vector parameters ex:paramA and ex:paramB have uncertainties given by the covariance matrices ex:covA and ex:covB, respectively. The uncertainties of the vector parameters are given by diagonal matrices as the parameters $a_{i}$ and $b_{i}$ are uncorrelated.

**Figure 8:**
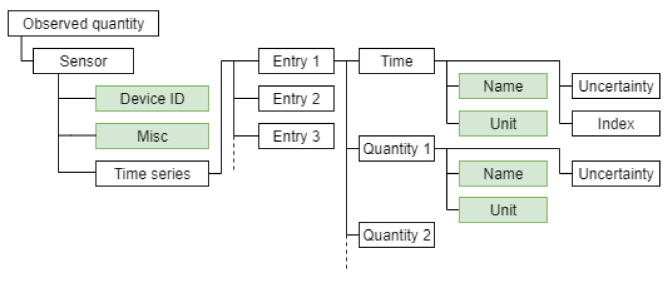
Abstract representation of a time series corresponding to data from a given sensor. The metadata necessary to interpret the actual measured quantities and time instances is contained in the scheme. The fields with green shading correspond to the metadata fields.

In our practical implementation, as described in Section 3, we handle numerical data and its semantic description separately. The raw numerical data coming from the sensor is handled using the Python package time-series-buffer. This allows us to store value, value uncertainty, timestamp and timestamp uncertainty in an efficient buffer structure on an FIFO basis. The sensor’s semantic self-description is handled using the Python package time-series-metadata. This package directly covers the core metrological aspects of a measurement value: time name, time unit, quantity name, quantity unit and the sensor ID it is originating from. Further aspects about the sensor are currently less standardized, but can be stored within a miscellaneous “misc” object. The basic structure of the time-series-metadata object is illustrated in Figure 8. This object could store metadata of the type given in Figure 7 generated using the ontology proposed by [21]. Metadata corresponding to the sensor calibration information can be obtained from official metrological documentation (e.g., a digital calibration certificate [30]) or datasheets. A more semantic approach to represent common mathematical models will be the focus of further research.

Additional attributes that track modifications of the data stream by agents are also possible. The description of the quality of data (QoD) sent from or received by a sensor is of particular interest. In general, the QoD depends on the principle of measurement used, the provenance of the data and the internal processing performed on it as well as the external environmental conditions at the time of measurement. A method to automate the process of enriching a data stream with quality semantics using an instantiation of the SSN ontology was proposed in [31]. By enriching sensor data with semantic quality information, it would be possible for higher logical agents to retrieve sensor data that satisfies given quality criteria such as accuracy, precision and drift. It would additionally be possible to ascribe quality metrics to data based on sensor properties such as resolution, response time, sensitivity, etc. Self-describing sensors can also enable higher logical agents to group connected devices to form networks and clusters based upon provided semantic information. As an example, consider location information. Given the ontological support, a relationship between two given device locations can be evaluated. This could be the estimated delay between two sensor locations in a production process or simple “is neighbor” information of floor tiles in an intelligent shop floor [32]. Such a grouping of connected devices can also help in exploiting the benefits of redundancy in a sensor network. For instance, semantic information can be used to generate more robust measurements from a disparate sensor group. A more detailed discussion of redundancy in sensor networks and its implications will be presented in the subsequent section.

## 5. Benefits of Smart Traceability and Redundancy in Sensor Networks

In Section 3, it was shown how a sensor can be extended by an SUU to construct a smart sensor in the industrial IoT. This smart sensor can store different types of metadata (like uncertainty information, location, etc.), which enables the SUU to provide much more machine-readable and machine-interpretable information regarding the measurement than the classical data pair (timestamp, sensor value). How this semantic information can be studied, modeled and stored was discussed in Section 4. One possible application of using machine-interpretable information is that the network may become aware of *redundancy* present in the network. In networks with a measurement task, i.e., where the goal is to infer the value of a measurand (the quantity to be measured), redundancy can be defined as the property that there are multiple, independent ways of deriving the value of the measurand from the set of measured sensor values. Two general advantages of redundancy are that it makes the network more robust and resilient against failing sensor nodes. In this section, some other advantages of redundancy relating to metrology and smart traceability will be discussed.

In metrological sensor networks, measurements made by sensor nodes are traceable and their measured values are accompanied by an uncertainty statement. In an agent framework like the agentMET4FOF framework being developed in [1], a particular agent can be in charge of combining data streams and performing uncertainty calculations. If a smart data analysis agent (SDAA) finds out that *m* nodes in the network are redundantly measuring the same quantity *Y* based on the semantic information provided by the smart sensors, then the following happens. The *m* different estimates $y_{1}$, $y_{2}$, …, $y_{m}$ of the measurand *Y* with associated uncertainties $u(y_{1})$, …, $u(y_{m})$ can be combined by the SDAA by calculating an uncertainty weighted averaged estimate $\hat{y}$ of the measurand with associated uncertainty $u(\hat{y})$, i.e.,:

(2) $$\begin{array}{ccc} u(\hat{y}) & = & {\left(\sum_{i=1}^{m}\frac{1}{u^{2}\left(y_{i}\right)}\right)}^{-1/2} \end{array}$$

(3) $$\begin{array}{ccc} \hat{y} & = & u^{2}\left(y\right)\sum_{i=1}^{m}\frac{y_{i}}{u^{2}\left(y_{i}\right)}. \end{array}$$

This calculation is the same as what can be used in the context of evaluating laboratory intercomparisons [33]. The knowledge of individual sensor uncertainties allows the optimal usage of the available information. Note that correlations between different estimates can (and should) be taken into account as well in the calculations. If the uncertainty (covariance) matrix of the $y_{i}$ (1≤i≤m) is given by $V_{y}$, and $y={\left(y_{1},\ldots ,y_{m}\right)}^{T}$ and $e={\left(1,\ldots ,1\right)}^{T}$ are column vectors of length *m*, then the best estimate and associated uncertainty follow from

$$\begin{array}{ccc} u(\hat{y}) & = & {\left(e^{T}V_{y}^{-1}e\right)}^{-1/2}\\ \hat{y} & = & u^{2}\left(\hat{y}\right)\;e^{T}V_{y}^{-1}y. \end{array}$$

The amount of redundancy “RedUnc(*k*)” ([34]) in a network for a specific measurement task can be quantified by assessing how much the expanded measurement uncertainty of the measurand maximally increases when leaving out *k* arbitrary sensor(s) from a network with *n* sensors. This can be done until the maximum possible number $K_{0}$ of sensors that can be left out has been reached while it is still possible to calculate the value of the measurand. This latter number $K_{0}$ is called “RedExcess” in [34]. Let $U_{k}$ denote the maximum expanded uncertainty when evaluating *Y* using n−k sensors, with the maximum taken over all subsets with n−k sensors. The redundancy metric RedUnc(*k*) and its relative version RedUncRel(*k*) are given by

$$\begin{array}{ccc} \mathrm{RedUnc}(k)=U_{k}-U_{0} & \mathrm{and} & \mathrm{RedUncRel}(k)=\frac{U_{k}-U_{0}}{U_{0}}\cdot 100\%. \end{array}$$

These redundancy metrics are a function of *k*. They can be summarized by averaging the uncertainty increase per removed sensor per total number of sensors taken out until a maximum number *K*. This is called the *redundancy loss* of the network, “RedLoss${}_{K}$”. *K* can be chosen equal to $K_{0}$ or less. In formula form this gives

$$\begin{array}{ccc} {\mathrm{RedLoss}}_{K} & = & \frac{1}{U_{0}}\frac{1}{K}\sum_{k=1}^{K}\frac{U_{k}-U_{0}}{k}\cdot 100\%. \end{array}$$

In the case of a redundancy loss of 0%, there is no increase of uncertainty when up to *K* sensors are taken out from the network and the network is perfectly redundant. A redundancy loss of 20% means that the expanded uncertainty of the measurand increases on average by 20% per removed sensor.

**Figure 9:**
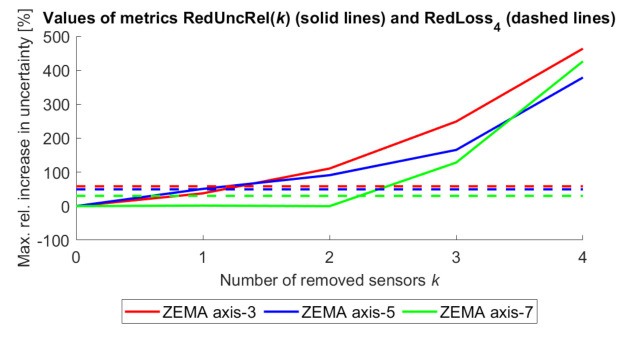
Relative uncertainty increase and redundancy loss values for condition monitoring of EMCs in ZeMA testbed when using 5 sensors and taking out 0 to 4 sensors.

As a practical example, the RedUnc(*k*) and RedLoss${}_{K}$ metrics were calculated for a data set produced by the ZeMA testbed for condition monitoring of an electromechanical cylinder (EMC) [10]. The EMC was measured by 11 sensors measuring different quantities like electrical current, vibrations and pressures. A trained machine learning algorithm can predict the residual lifetime of the EMC based on the signal of any single sensor. As it could not be founded on an uncertainty evaluation of the sensor measurements, the uncertainty of the estimates was quantified in a heuristic way using the prediction error, together with uncertainty propagation through the model with the addition of a model error. When using all sensors, the estimate has the lowest prediction uncertainty. When taking out 1, 2, …sensors, the maximum uncertainty increases (unless the uncertainty calculation depends on the data values themselves for some cases). Both the relative uncertainty increase as a function of *k* and the overall redundancy loss value (constant) are shown in Figure 9 for the case when 5 sensors are used and 0 to 4 sensors are removed. The redundancy loss for the EMCs varies between 30 and 60%.

Another advantage of traceable, redundant smart sensor networks is that incorrect sensor values can be more easily detected and removed. If a quantity is measured at least twice, a statistical test can be performed to assess if the measured values are consistent in view of the specified uncertainties. If this is not the case, a warning can be raised to the user. A test that is often used in a metrological context [33] is based on the observed chi-squared value

$$\begin{array}{ccc} \chi_{\mathrm{obs}}^{2} & = & \sum_{i=1}^{m}{\left(\frac{y_{i}-\hat{y}}{u^{2}\left(y_{i}\right)}\right)}^{2}. \end{array}$$

Assuming that the uncertainties of the $y_{i}$ are normally distributed, the distribution of $\chi_{\mathrm{obs}}^{2}$ is chi-squared with m−1 degrees of freedom. As a consistency check it can be verified if the probability of observing a value of $\chi_{\mathrm{obs}}^{2}$ or more is less than 5%, in which case the individual sensor estimates are evaluated as being inconsistent.

If a quantity is measured at least three times (m≥3), a smart algorithm can determine the largest consistent subset (LCS) of measured values. In the case that two values are correct, and one sensor value is incorrect, the algorithm can identify and reject the incorrect value and return a best estimate based on the consistent values. See [33], where this method is applied in the context of interlaboratory comparisons. This idea can be extended to situations in which the quantity of interest is not directly measured (e.g., a temperature being measured by multiple sensors) to the case when there is a (linear) model y=a+Ax relating a vector of sensor values x to a vector y of multiple estimates of the measurand *Y* (see ZeMA example below). In the latter case a modified algorithm to find the largest consistent subset of sensor values (LCSS) can be used ([35]). The main steps of the algorithm are outlined in Algorithm 1, where $V_{x}$ denotes the uncertainty matrix of x. The algorithm always terminates, because a subset with one sensor always provides a consistent estimate.

As a practical application this algorithm was applied to the ZeMA testbed for condition monitoring of an EMC [10]. Using 11 separate machine learning models for each sensor, these 11 models sometimes give different predictions for the residual lifetime at a given set of measurement data taken at a specific time point. When an uncertainty estimate is associated with each estimate, the LCS algorithm can be applied to the different predictions. When uncertainty information of the raw sensor measurements is known and a linear machine learning model is used, the LCSS algorithm can even be applied to the raw sensor values. In this way a new, metrologically underpinned estimate can be calculated, where inconsistent estimates or sensor values have been identified and rejected. For the ZeMA data set example, the results of LCS, LCSS and other data analysis approaches were of comparable quality.

**Algorithm 1:** Outline of largest consistent subset of sensor values (LCSS) algorithm.

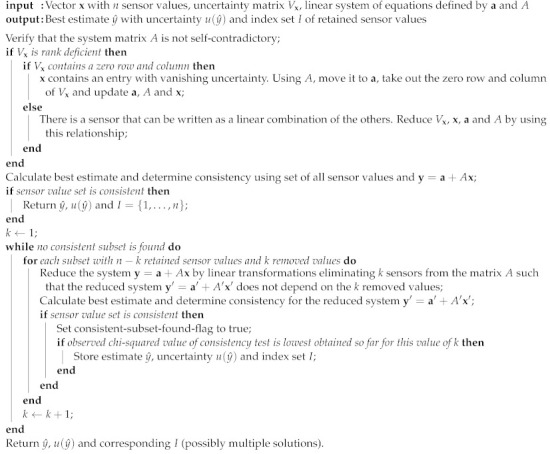



In a smart traceable sensor network the replacement of sensors is more straightforward: As the response of a sensor is well characterized and all deviations are known, these deviations can be uploaded to and corrected by the software of digital smart sensors. In the case of a strongly redundant network, a new sensor can be calibrated and/or characterized by comparing it with the values indicated by sensors which are known to be strongly correlated with the new sensor. For some applications there may be time periods when all sensors are supposed to give equal results, e.g., a uniform temperature in a production hall during nighttime, which can be exploited to get a form of “smart traceability”. Another observation is that for some applications a full sensor calibration is not needed. In the ZeMA testbed example the constructed machine learning models only use the sensor amplitudes at very specific frequencies. Smart traceability may involve only calibrating these amplitude responses and not addressing phase information or other frequencies. As the ZeMA testbed contains three current sensors measuring different current phases, it can also be envisaged to calibrate a new current sensor by putting it in series with one of the existing sensors and comparing the responses. Another option is to verify if the amplitudes of the frequencies of interest are almost identical in the three current phases, possibly only in certain periods of time. In that case a new sensor can directly be calibrated in situ for the amplitude response at these frequencies. If the network is redundant, sensors can often be replaced without having to shut down the complete network first (if not prevented by, e.g., safety precautions).

We thus see that redundancy in industrial networks is a powerful tool for a large range of purposes. This extends from being resilient to broken sensor nodes, being able to identify and reject incidentally faulty sensor values, reducing measurement uncertainty, replacing sensors on the fly without having to shut down the complete network and allowing the implementation of smart calibration strategies in order to reach smart traceability. When the sensors are smart and semantic meta information is encoded in a machine-interpretable fashion, smart data agents can perform the required network meta-analysis (e.g., establishing the presence of redundancy) in an automatic way, and take advantage of the outcomes of the meta-analysis where possible.

## 6. Conclusions and Outlook

Metrology for the Internet of Things (IoT) and similar concepts of heterogeneous sensor networks is of growing interest, but many challenges remain to be solved. These include the requirement of novel calibration approaches for smart digital sensors, mathematical modeling of complex sensor networks for uncertainty propagation and uncertainty-aware machine learning methods.

The traceability of measurements to the SI units is important also in the IoT. Even more so as the data analyses become automated and intelligent, and as the layout and topology of the network can change rapidly. The efficient application of artificial intelligence (AI) methods to a sensor network requires a machine-interpretable representation of the knowledge available about the individual measuring instruments and their relation to each other. Smart traceability in the sense described in this paper can help to achieve that. Furthermore, the replacement or failure of a single sensor must not result in the necessity to re-train large parts of the AI method. With traceable measurements, this can be avoided by taking the calibration information into account in the data analysis. Traceability also allows more efficient data analysis methods to be applied, for instance, to take advantage of network redundancy. Methodologies like that of the largest consistent subsets can be applied to determine sub-networks of smaller measurement uncertainty—and thus, with more reliable results.

Many of these principles and concepts have been applied by metrologists in science and industry for decades. However, with sensors becoming smart and sensor networks becoming more complex, the situation is changing significantly. Thus, the metrology principles such as traceability and measurement uncertainty evaluation need to become smart too. They also need to become applicable to complex situations in a highly automated way. The agent framework approach described here can help to achieve this. With a semantic description of the sensors and the sensor network, machine-readable information is available that allows algorithms to process the data streams automatically. In this way, the principles applied by metrologists off-line and manually can be implemented in algorithms for automated processing.

Future work will contain the end-to-end implementation of these principles. As a first step, the existing agent framework implementation will be expanded into a simulation toolbox for metrological principles in the IoT.

## Data Availability

All data used in this publication is publicly available. Corresponding references are given in the paper.
